# How surface acting affects turnover intention among family doctors in rural China: the mediating role of emotional exhaustion and the moderating role of occupational commitment

**DOI:** 10.1186/s12960-023-00791-y

**Published:** 2023-01-26

**Authors:** Anqi Wang, Changhai Tang, Lifang Zhou, Haiyuan Lv, Jia Song, Zhongming Chen, Wenqiang Yin

**Affiliations:** 1 School of Public Health, Weifang Medical University, Weifang, China; 2 School of Public Affairs, Zhejiang University, Hangzhou, China; 3 School of Business, NingboTech University, Ningbo, China; 4 School of Management, Weifang Medical University, Weifang, China

**Keywords:** Family doctors, Surface acting, Emotional exhaustion, Occupational commitment, Turnover intention

## Abstract

**Background:**

Family doctors in rural China are the main force for primary health care, but the workforce has not been well stabilized in recent years. Surface acting is an emotional labor strategy with a disparity between inner feelings and emotional displays, provoking negative effects such as emotional exhaustion, occupational commitment reduction, and, consequently, increasing turnover rate. With the Conservation of Resources theory, this study explores how the surface acting of rural family doctors affects turnover intention through emotional exhaustion and investigates what role occupational commitment plays in this relationship.

**Methods:**

With a valid response rate of 93.89%, 953 valid data were collected by an anonymous self-administered questionnaire survey in December 2021 in Shandong Province, China. Cronbach’s Alpha and confirmatory factor analysis (CFA) were used to estimate reliability and construct validity, respectively. The PROCESS macro in SPSS was performed to analyze the mediating and moderated mediation effects of surface acting, emotional exhaustion, occupational commitment, and turnover intention.

**Results:**

Reliability and validity indicated that the measurement instruments were acceptable. Surface acting had a direct positive effect on turnover intention (*β* = 0.481, 95% CI [0.420, 0.543]). Emotional exhaustion partially mediated the effect of surface acting on turnover intention (indirect effect: 0.214, 95% CI [0.175, 0.256]). Occupational commitment moderated the effect of emotional exhaustion on turnover intention (*β* = − 0.065, 95% CI [− 0.111, − 0.019]), and moderated the indirect effect of surface acting on turnover intention via emotional exhaustion (index of moderated mediation: − 0.035).

**Conclusions:**

Emotional exhaustion partially mediates the relationship between surface acting and turnover intention among family doctors in rural China, and occupational commitment moderates the direct effect of emotional exhaustion on turnover intention and further moderates the mediating effect. Policymakers should pay more attention to the effects of emotional labor and emotional resource depletion on the stability of rural health human resources.

## Introduction

The family doctor services policy is designed to play the role of “gatekeeper” for family doctors in primary health care, providing residents with an initial diagnosis and comprehensive and coordinated services throughout the life cycle [1]. This policy has been implemented in more than 50 countries, including the United States, the United Kingdom, Germany, and Canada, and has been proven in practice to be a cost-effective and feasible way to protect and maintain public health [2]. Although operational mechanisms and service models of the family doctor services policy are varied among countries, there are some common features and practices that require residents and family doctors to sign service contracts. For example, residents in the Netherlands need to choose a single general practitioner to register with [3], general practitioners in Norway have to sign healthcare contracts with residents [4], and the UK has adopted a state-managed model requiring residents to sign contracts with family doctors [5].

In 2016, the Chinese government comprehensively implemented the family doctor contract services system, intending to improve the health status of the population and the ability of patients to rationally choose medical institutions [6]. The family doctor contract services system in China works through residents voluntarily signing service contracts with family doctor teams, who provide free basic medical and public health services, as well as paid personalized health management services to help residents obtain long-term coordinated healthcare [7]. The family doctor team in China is generally composed of family doctors, nurses and public health doctors [8]. Family doctors are usually general practitioners registered in primary medical institutions and competent rural doctors, who play the role of leaders in teams and mainly take care of common diseases, provide consultation and referral services according to patients’ conditions, conduct interventions and follow-ups for patients with chronic diseases, perform home visit services for patients with special needs, etc. The main duties of nurses in family doctor teams are to understand the basic conditions of contracted residents, and assist family doctors in carrying out various services. Doctors engaged in preventive care, health education, and other public health services serve as public health doctors in family doctor teams, who are responsible for monitoring the health status of contracted residents in real-time, establishing and updating their health records, providing health education and consultation to contracted residents, and developing health plans for them through discussions with family doctors, etc. [8, 9]. The effective implementation of the family doctor contract services system is conducive to improving the quality of primary health care in China, accelerating the development of the graded diagnosis and treatment system, and ensuring targeted medical services to patients in a minimum amount of time [10].

In China, rural areas are defined as geographical complexes with natural, social, and economic characteristics and multiple functions such as production, living, ecology, and culture that are outside urban built-up areas, including towns and villages [11]. According to the 7th National Census of China in 2020, the rural population was approximately 500 million, accounting for 36.11% of the total population [12]. Studies have shown that rural residents in China have urgent medical demands, with an average of only 1.3 licensed doctors per 1 000 rural residents [13, 14]. A growing number of rural family doctors in China play an indispensable role in the provision of primary health care [15]. However, a heavy workload and great work pressure can lead to their turnover intention.

Turnover intention is possibly the most important and direct antecedent to the turnover decision, and the most immediate predictor of turnover [16], which affects the stability of the family doctor pool and the efficiency of services provided [17]. Most perspectives have placed turnover intention as the negative outcome of the combined evaluation of job satisfaction and the utility of the current job, and further taking steps to secure future employment, which can trigger the actual turnover [18]. In China, family doctors who are the main force of primary health care also suffer from continuous workforce shortages and high levels of turnover intention [19]. For example, Gan et al. [20] surveyed 3 236 family doctors in China who were registered general practitioners in primary medical institutions and found that the percentage of those with moderate or high levels of turnover intention was 71.1%. Li et al. [21] revealed that younger family doctors reported higher levels of turnover intention, with 80% of family doctors under 35 years of age having turnover intention. Family doctors with turnover intention result in lower morale, hidden absenteeism, and poor performance [22]. The prevalence of chronic diseases is higher and the accessibility of medical resources is lower in rural China [13, 23]. Widespread and continuous turnover intention of rural family doctors could aggravate the shortage of human resources, undermine the stability and sustainability of the healthcare system, and reduce the quality of primary health care, all of which are detrimental to improving the health status of rural residents.

Mobley et al. [24] identified job content characteristics as one of the significant factors influencing individuals’ turnover intention. Numerous studies [25–27] have also confirmed that the job characteristics of Chinese family doctors, such as heavy workload, variety of work, limited opportunities for career advancement, and the discrepancy between effort and income, can lead to turnover intention. A further study [21] has found that 90% of Chinese family doctors feel constantly fatigued and emotionally exhausted, which is not only related to the mentioned job characteristics, but also, more importantly, to the significant investment of energy and emotional resources. The accomplishment of family doctors’ work goals is closely related to the cooperation of residents. In this regard, family doctors keep optimizing their services to obtain residents’ support, including constantly improving their communication and attitudes to meet residents’ emotional demands, which implies more emotional labor on the part of family doctors. Surface acting, as an emotional labor strategy with a disparity between inner feelings and emotional displays, increases family doctors’ emotional depletion and work stress, resulting in emotional exhaustion and mental fatigue, and thus turnover intention. Additionally, occupational commitment, as a measure of individuals’ affective response to their occupation, can replenish the depletion of emotional resources and can also play a role in turnover intention [28].

This study draws on the Conservation of Resources (COR) theory to examine the relationships between surface acting, emotional exhaustion, occupational commitment, and turnover intention among family doctors in rural China. The COR theory is a resource-based stress theory that assumes that absolute or relative decreases in resources can lead to increases in individual stress, with the basic tenet that “individuals strive to obtain, preserve, foster, and protect the resources they value” [29]. The COR theory proposes five principles of “primacy of loss, resource investment, gain paradox, resource desperation, resource caravans and passageways” to explain individual resource conservation responses in coping with stress [30]. The emotional resource is an individual resource, and the COR theory suggests that individuals’ resources are depleted and not replenished at work, resulting in stress and emotional exhaustion due to resource imbalance [31]. Surface acting is an emotional labor strategy in which individuals deliberately fake their true inner feelings to project the emotional display expected by organizations, and it depletes the individuals’ emotional resources in the process [32]. According to the COR theory, when emotional resources are exhausted, individuals can, on one hand, promote the rapid recovery of emotional resources by timely replenishment of intrinsic resources through occupational commitment, which is of great value to the individual. On the other hand, it is also possible to activate individuals’ self-protective defensive mechanisms and show some aggressive and irrational behaviors, such as leaving the job, in order to protect their remaining emotional resources. This study explored the above issues by analyzing a group of rural family doctors, in the hope that the findings of this study can provide important practical guidance for health administration departments and medical institutions as a basis for developing interventions to reduce the turnover rate of family doctors. Meanwhile, this study is also beneficial to break through the limitations of existing studies, which mostly explore the workload and work rewards of family doctors, with little consideration of the amount of emotional labor undertaken by family doctors in their actual work, especially the insufficient awareness of the adverse effects of surface acting.

## Literature review and hypothesis development

### Surface acting and turnover intention

The work of doctors requires not only physical and mental effort, but also emotional labor [33]. Emotional labor, regulating the expression of feelings at work, was defined by Hochschild [34] as the management of feelings for creating a publicly observable display of the face and body to get paid. Emotional labor, a form of labor different from mental and physical labor, involves following specific emotional display rules that refer to the standards set by organizations as the appropriate expression of emotions, and requires employees to consciously control their emotions to achieve tasks regardless of how they truly feel [32]. Deep acting and surface acting are two main strategies of emotional labor, but they differ in the effort and intention to regulate emotional expressions. When engaging in deep acting, individuals attempt to change their inner feelings and show empathy to match the desired emotional displays [35]. However, in surface acting, individuals display emotions via faking without shaping inner emotional states in order to cope with the work, which is assumed to be “faking in bad faith” [36].

Family doctors are in extensive contact with residents in their daily work. Given the low health literacy of rural residents [37], and poor trust of patients toward doctors [38], family doctors in rural areas are more frequently required to employ emotional strategies to improve communication and attitudes in order to obtain the cooperation of rural residents [39]. It has been revealed that deep acting is gradual over time and doctors rarely change their feelings immediately to truly understand their patients, but instead, surface acting is employed by doctors to help patients develop hope and change viewpoints [40]. Moreover, a positive relationship between harsh treatment from patients and surface acting by doctors has been reported [39]. Gan et al. [17] found that Chinese family doctors are regularly exposed to workplace violence, with emotional abuse from patients being the most common type, so family doctors often employ surface acting in their work. Meanwhile, taking into account the demographics and health literacy of rural residents, family doctors in rural areas are more inclined to control their inner feelings and cater to the medical demands of the patients by surface acting to accomplish their work targets.

Compared to authentic, positive emotions elicited by deep acting, surface acting is generally related to more detrimental outcomes, such as developing emotional dissonance and causing emotional exhaustion [41]. Grandey [42] found that individuals who have to engage in high levels of surface acting are more likely to have turnover intention. Evidence of the link between surface acting and turnover intention can be provided from theoretical studies on emotional dissonance. Emotional dissonance, a state of being uncomfortable because of a discrepancy between individuals’ feelings and their emotional displays, often occurs as individuals engage in surface acting, and individuals are then motivated to remove themselves from the surface acting that has caused the emotional dissonance, resulting in their turnover intention [43]. Rural family doctors suffer from emotional dissonance because of their surface acting and have turnover intention to get rid of this state early. Studies in the medical field have further indicated that professionals who regularly engage in surface acting have high levels of turnover intention [44, 45]. Summarizing the above theoretical arguments and empirical findings, we predict the following:

#### **Hypothesis 1**

Rural family doctors’ surface acting is positively related to turnover intention.

### The mediating role of emotional exhaustion

Emotional exhaustion, an essential component of job burnout, is a chronic state where employees are depleted of emotional and physical resources due to excessively strict job requirements and continuous work stress [46]. The Conservation of Resources (COR) theory asserts that resources are available to help individuals cope with stressful experiences, but are also typically depleted by confronting stressful circumstances, and it further predicts that resource loss is a dominant component of the stressful process [47]. Resources refer to objects, personal characteristics, conditions, or energies that are valued by individuals or served as a means of attaining them, of which emotional resources also matter to individuals [30]. Emotional exhaustion occurs when individuals perceive they no longer have sufficient emotional resources to cope with the stresses being faced, or perceive their resources to be threatened with loss and not available after being invested [48].

Family doctors are regularly exposed to emotional happenstances and are expected to accept emotional management as part of their basic duties [49], and surface acting is a common strategy employed by them. In this circumstance, rural family doctors are constantly exposed to excessive mental stress as they fake their inner feelings in surface acting. According to the COR theory, the emotional resources depleted by surface acting can increase stress, and emotional exhaustion occurs when rural family doctors’ emotional resources cannot overcome mental stress, and thus surface acting is positively associated with emotional exhaustion [31]. Numerous studies [50, 51] have also consistently revealed that surface acting serves as an antecedent to emotional exhaustion. A study of Chinese hospital nurses by Deng et al. [52] found that surface acting is positively associated with emotional exhaustion. Zhao et al. [53] also confirmed in a study of front-line service teams that surface acting positively predicts emotional exhaustion. With this theoretical and empirical evidence, we expect the following:

#### **Hypothesis 2**

Rural family doctors’ surface acting is positively related to emotional exhaustion.

Prior studies [48, 54] have linked emotional exhaustion to numerous adverse work outcomes, such as increasing turnover intention. A study [55] with general practitioners in China confirmed a positive association between emotional exhaustion and turnover intention. A meta-analysis by Kim et al. [56] on predicting factors of turnover intention for Korean hospital nurses also identified emotional exhaustion as a significant factor contributing to turnover intention. Shah et al. [57] conducted a study of nurses’ job stress during the COVID-19 pandemic to support the positive effect of emotional exhaustion on turnover intention. The relationship between emotional exhaustion and turnover intention can also be explained by the COR theory. The basic tenet of the COR theory states that individuals strive to acquire, retain, and protect what they value, and minimize the threat of any resource loss, with the absence of resources leading to defensive attempts to protect the remaining resources [29]. This theory can be taken to explain that when faced with emotional exhaustion, individuals choose to leave their jobs to protect their remaining emotional resources from continuing to deplete, thus minimizing the loss of their emotional resources [32].

Compared to urban areas, rural areas are relatively lacking in terms of healthcare facilities construction and physician team building [58]. Family doctors in rural areas have heavy workloads with workforce shortages, and more frequent emotional labor [17], resulting in a state of emotional exhaustion where resources are often depleted and difficult to preserve. As explained by the COR theory [32], individuals’ self-protective defensive mechanisms are triggered in the state of emotional exhaustion, and leaving can protect their remaining emotional resources from further depletion. Therefore, rural family doctors in this situation may develop turnover intention, by leaving the present job to keep them from the threat of excessive resource depletion. Thus, the following hypothesis is proposed:

#### **Hypothesis 3**

Rural family doctors’ emotional exhaustion is positively related to turnover intention.

The COR theory suggests that individuals’ initial loss of resources will cause further loss with adverse behaviors and effects in the future, resulting in what is called a “downward spiral of loss” [59]. The COR theory and its “loss spiral” account for the fact that rural family doctors’ continuous surface acting accelerates the loss of emotional resources, leading to emotional exhaustion and further adverse behavioral or attitudinal inclinations toward avoiding or leaving the current situation. Thus, emotional exhaustion is a mediating role of surface acting and turnover intention, as confirmed in relevant studies [60, 61]. Therefore, the following hypothesis is proposed:

#### **Hypothesis 4**

Emotional exhaustion mediates the relationship between rural family doctors’ surface acting and turnover intention.

### The moderating role of occupational commitment

Occupational commitment is defined in one dimension as affective commitment, expressing individuals’ positive feelings about their occupation, and in multiple dimensions such as continuance commitment and normative commitment [62, 63]. Yin et al. [64] described Chinese physicians’ occupational commitment from the one-dimensional perspective as positive feelings toward the occupation, expressing enjoyment, affirmation, and identification. Occupational commitment is perceived as a personal inner motivation for the occupation [65]. Individuals with strong occupational commitment are more adept at motivating their inner resources to properly solve problems at work, which can play an important role in the intention to stay [66]. Consistent with the resource replenishment principle in the COR theory [67], which states that individuals rely on additional resources to replenish lost resources and minimize the adverse impact of resource loss, occupational commitment allows individuals to reinforce personal characteristic resources, including resilience, in order to enhance their mental strength when dealing with work stress and resource depletion and strengthen their ability to adapt to challenging environments, thereby reducing the impact of an adverse events [29, 68]. A study of Chinese village doctors [14] has shown that improving resilience can reduce turnover intention.

Emotional exhaustion is the resulting state of an excessive depletion of individuals’ emotional resources and can occur from an undesirable work situation that requires individuals to proactively respond to [69]. When occupational commitment is relatively high, it provides a strong mental resource for individuals to reduce the effect of emotional exhaustion on turnover intention. When occupational commitment is relatively low, individuals cannot develop solutions or personal resources, thus making the effect of emotional exhaustion on turnover intention more severe. This logic is supported by studies indicating that occupational commitment can reduce the effect of emotional exhaustion on turnover intention. For example, it has been confirmed that higher levels of occupational commitment are less likely to involve emotional exhaustion, which can also reduce the adverse effect of emotional exhaustion, such as turnover intention [70, 71]. In addition, occupational commitment is a predictor of turnover intention, with higher levels of occupational commitment contributing to lower turnover intention [16, 62]. Combining the above analysis, and considering the existing rural health system situation, we assume that rural family doctors with higher levels of occupational commitment are less likely to have turnover intention after exposure to emotional exhaustion. Therefore, the following hypothesis is formed:

#### **Hypothesis 5**

Occupational commitment moderates the positive relationship between rural family doctors’ emotional exhaustion and turnover intention, such that the relationship is weaker for those with a higher level of occupational commitment than with a lower level of occupational commitment.

The above hypotheses demonstrate an indirect effect of rural family doctors’ surface acting on their turnover intention mediated by emotional exhaustion, while occupational commitment moderates the effect of emotional exhaustion on turnover intention. It is reasonable to integrate these hypotheses into a moderated mediation model, as we hypothesize as follows:

#### **Hypothesis 6**

Occupational commitment moderates the indirect effect of rural family doctors’ surface acting on turnover intention via emotional exhaustion, such that the indirect effect is weaker for those with a higher level of occupational commitment than with a lower level of occupational commitment.

## Methods

### Study design and sampling

Participants in this study were family doctors in rural China. Each participant read a statement explaining the purpose of the survey and consented to be involved in this survey. Before the formal survey, we conducted a pilot test in July 2021 using random sampling, and 150 questionnaires were distributed and 134 valid questionnaires were collected, with the valid response rate of 89.33%. In the pilot test, the mean age of rural family doctors was 48.36 years old, with 50.75% being female, and the reliability of the questionnaire was 0.668. Data for the final analysis of this study were obtained from a baseline survey of family doctors in rural areas held in December 2021 in Shandong Province.

Shandong Province is the second most populous province in China, with a population of 101.70 million people in 2021, of which 36.06% population lived in rural areas [23, 72]. Shandong Province is also an economically developed region in eastern China, with a GDP of 1147.13 billion US dollars in 2021 [72]. Shandong Province currently has 16 prefecture-level cities (a prefecture-level city is an administrative level below the province and above the county). Shandong Province issued the *Guidelines for Family Doctor Contract Services in Shandong Province* in 2017, in line with the guidelines required by the State Council, and implemented family doctor contract services based on this guideline with good achievements. By the end of 2021, Shandong Province formed a total of 32 000 family doctor teams and had 46.86 million contracted residents [72].

We conducted this formal survey using a stratified random sampling method. First, three prefecture-level cities in Shandong Province were sampled according to economic level, representing economically developed cities, economically moderate cities, and economically underdeveloped cities. Then, three counties were randomly sampled in each prefecture-level city, four to six townships were sampled in each county according to the size of the population, and twenty to thirty family doctors were randomly sampled in each township for the anonymous self-administered questionnaire survey. We assigned three to five well-trained investigators at each survey site to provide face-to-face assistance if participants had any questions when filling out the questionnaire. Each questionnaire was reviewed for quality by at least one investigator once it was completed. The investigators manually checked each item of every completed questionnaire to avoid omissions, mistakes, and logical errors. For example, if a rural family doctor wrote his/her family yearly income as 200 US dollars, which is far less than the average of Chinese family yearly income, the investigator would re-interview the participant and ask him/her to correct it on the spot if there was a filling error. Or if the same value was selected for all answers to the measured variables in the questionnaire, the investigator would ask the participant to reconfirm their accuracy of the answers. A total of 953 valid questionnaires were used for analysis after excluding those with incomplete data on key variables, with a valid response rate of 93.89%.

### Measurement instruments

We measured the variables and designed the questionnaire according to the hypotheses of this study. The questionnaire covered the scales of surface acting, emotional exhaustion, occupational commitment, and turnover intention. All items were rated on a five-point Likert scale, with a range from 1 (strongly disagree) to 5 (strongly agree).

Surface acting was measured using five items reported by Wu [73], which was translated and revised from the scale of surface acting adopted by Grandey [36]. Sample items include “I can pretend to be in a good mood at work, even if I am not inside” and “It is like acting for me to show the appropriate expression and attitude at work”. These items have been verified to be of good reliability and validity in the sample of the Chinese population [74, 75]. Cronbach’s Alpha for our sample was 0.846.

Emotional exhaustion, an essential dimension of burnout, was measured with a five-item scale reported by Li et al. [76]. This scale was adapted from the Maslach Burnout Inventory-General Survey (MBI-GS) [77]. Sample items include “I feel physically and mentally exhausted from work” and “I feel exhausted when I finish work every day”. Studies have confirmed the good reliability and validity of this scale in the Chinese population [78, 79]. Cronbach’s Alpha for our sample was 0.934.

An adaptation of Huang and Yin’s [64] six-item scale of occupational commitment was used to measure how committed employees were to their occupation. An example item is “I am proud of my occupation”. The scale has been widely available in Chinese samples, showing good reliability and validity [80]. Cronbach’s Alpha for the present sample was 0.926.

Turnover intention was measured using a three-item scale, which translated by Yu et al. [81], and has been reported to have good reliability and validity in the Chinese population. A sample item is “I might be looking for a new job in the next year”. Cronbach’s Alpha for the present sample was 0.876.

### Statistical analysis

First, a descriptive analysis was conducted to describe the demographic characteristics. Then, confirmatory factor analysis (CFA) was performed to assess the construct validity of the measurement variables using Amos 21.0. Given the sensitivity of the Chi-square fit statistic to the sample size, the ratio of the Chi-square statistic to the respective degrees of freedom (*χ*^2^/*df*) and a variety of other fit indices have been proposed to assess model adequacy [82]. In this study, the validity of the measurement variables was assessed with fit indices including *χ*^2^/*df*, Comparative Fit Index (CFI), Tucker–Lewis Index (TLI), root mean square error of approximation (RMSEA), and standardized root mean square residual (SRMR). We determined an acceptable fit using the conventional cut-off criteria of *χ*^2^/*df* < 3, CFI > 0.90, TLI > 0.90, RMSEA < 0.08, and SRMR < 0.08 [83, 84]. In addition, the Chi-square difference values of the models were used to assess the discriminant validity. Average variance extracted (AVE) and composite reliability (CR) were applied to evaluate the convergent validity, where the values of AVE above 0.5 and CR above 0.7 indicated good convergent validity [83]. Furthermore, bivariate correlations between surface acting, emotional exhaustion, occupational commitment, and turnover intention were analyzed by Pearson correlation analysis. Finally, the process macro in SPSS was performed to analyze mediating and moderated mediation effects, where Model 4 and Model 14 were used, respectively. In Model 4, surface acting was taken as the independent variable, turnover intention as the dependent variable, and emotional exhaustion as the mediating variable between surface acting and turnover intention. In Model 14, occupational commitment was set as a moderating variable between emotional exhaustion and turnover intention. Gender, age, marital status, educational background, and family yearly income were used as control variables in Model 4 and Model 14. The significance level for all tests was determined as *P* < 0.05.

## Results

### Demographic characteristics

Participants in this study were family doctors in rural China. Among the 953 participants, 599 (62.85%) were men and 354 (37.15%) were women. The age range of participants was from 20 to 80 years old, with the majority in the 40–49 age group, and the mean age was 46.65 years. Of the valid sample, 917 (96.22%) were married, and 515 (54.04%) graduated from a secondary technical school. The average yearly family income was 7458.99 US dollars. The specific demographic characteristics of the sample are shown in Table 1.

**Table 1:** Demographic characteristics of rural family doctors in this survey (*n* = 953)

Variables	Frequency (*n*)	Percent (%)
Gender		
Male	599	62.85
Female	354	37.15
Age (mean ± SD) = 46.65 ± 7.68		
< 30	8	0.84
30–39	139	14.59
40–49	499	52.36
≥ 50	307	32.21
Marital status		
Married	917	96.22
Unmarried/divorced/widowed	36	3.78
Educational background		
No medical educational background	10	1.05
Secondary technical school graduates	515	54.04
Junior college degree or higher	428	44.91
Family yearly income (USD)		
< 2 760.00	69	7.24
2 760.00–5 519.86	325	34.10
5 520.00–8 279.86	294	30.85
≥ 8 280	265	27.81

### Validity of measurement variables

As shown in Table 2, the results of the CFA showed that the four-factor model (surface acting, emotional exhaustion, occupational commitment, and turnover intention) compared with three alternative models was fitted optimally and the fit indices were at an acceptable level (*χ*^2^/*df* = 2.944, CFI = 0.981, TLI = 0.976, RMSEA = 0.045, and SRMR = 0.058). In addition, the Chi-square difference values between the four-factor model and other models were statistically significant (∆*χ*^2^ > 10.83, *P* < 0.001), suggesting good discriminant validity [83]. Furthermore, the values of AVE for the four-factor model were acceptable, ranging from 0.543 to 0.682, and the values of CR were all above 0.7, ranging from 0.823 to 0.918. Finally, the result of Harman’s single-factor test showed that the variance explained by the first unrotated factor extracted was 45.52%, which was less than the empirical value of 50% [85], indicating low common method biases in this study.

**Table 2:** Confirmatory factor analysis (CFA) results

Model	*χ*^2^	*df*	*χ*^2^/*df*	∆*χ*^2^(∆*df*)	CFI	TLI	RMSEA	SRMR
Four-factor model	400.350	136	2.944	–	0.981	0.976	0.045	0.058
Three-factor model^a^	1 637.144	139	11.778	1 236.794 (3)^***^	0.890	0.865	0.106	0.121
Two-factor model^b^	2 151.911	141	15.262	1 751.561 (5)^***^	0.853	0.821	0.122	0.122
One-factor model^c^	2 453.434	142	17.278	2 053.084 (6)^***^	0.831	0.796	0.131	0.130

*CFI* Comparative Fit Index, *TLI* Tucker–Lewis Index, *RMSEA* root mean square error of approximation, *SRMR* standardized root mean square residual, *∆χ*^2^ Chi-square difference value, *∆df* degrees of freedom difference value

***P < 0.001

^a^This model combined emotional exhaustion and occupational commitment into one factor

^b^This model combined surface acting, emotional exhaustion, and occupational commitment into one factor

^c^This model combined surface acting, emotional exhaustion, occupational commitment, and turnover intention into one factor

### Bivariate correlations

The mean, standard deviation and correlation coefficient of variables are displayed in Table 3. Surface acting was positively related to emotional exhaustion (*r* = 0.514, *P* < 0.01) and turnover intention (*r* = 0.449, *P* < 0.01), and negatively related to occupational commitment (*r* = − 0.364, *P* < 0.01). Emotional exhaustion was positively related to turnover intention (*r* = 0.518, *P* < 0.01), and negatively related to occupational commitment (*r* = − 0.495, *P* < 0.01). Occupational commitment was negatively related to turnover intention (*r* = − 0.586, *P* < 0.01).

**Table 3:** Mean, standard deviation, and correlation coefficient of variables (*n* = 953)

Variable	Mean	SD	1	2	3	4
1. Surface acting	2.296	0.905	1			
2. Emotional exhaustion	3.074	0.942	0.514**	1		
3. Occupational commitment	2.684	1.000	− 0.364**	− 0.495**	1	
4. Turnover intention	2.158	0.975	0.449**	0.518**	− 0.586**	1

*SD* standard deviation

**P < 0.01

### Hypothesis testing

The results in Table 4 display that surface acting positively predicted turnover intention in the total effect (*β* = 0.481, *P* < 0.001), supporting Hypothesis 1. Surface acting positively predicted emotional exhaustion (*β* = 0.532, *P* < 0.001), supporting Hypothesis 2. Emotional exhaustion positively predicted turnover intention (*β* = 0.403, *P* < 0.001), supporting Hypothesis 3. The above analysis could support Hypothesis 4, and emotional exhaustion served as a partial mediating role because the direct effect of surface acting on turnover intention was statistically significant (*β* = 0.267, *P* < 0.001). Additionally, the method based on 5000 bootstrapped samples was adopted to further test the mediating effect, and the result was considered statistically significant (*β* = 0.214, *P* < 0.001), indicating that emotional exhaustion mediated the effect of surface acting on turnover intention, with Hypothesis 4 being supported again.

**Table 4:** Regression results for total, direct and indirect effects (*n* = 953)

Effect	Hypothesis	Estimate	SE	95% CI
Total effect	H1: surface acting → turnover intention	0.481***	0.031	[0.420, 0.543]
Direct effect	H2: surface acting → emotional exhaustion	0.532***	0.029	[0.475, 0.589]
	H3: emotional exhaustion → turnover intention	0.403***	0.033	[0.339, 0.467]
	Surface acting → turnover intention	0.267***	0.034	[0.200, 0.333]
Indirect effect	H4: surface acting → emotional exhaustion → turnover intention	0.214***	0.021	[0.175, 0.256]

Covariates controlled (gender, age, marital status, educational background, and family yearly income) in the modeling analysis were found to be statistically insignificant

*SE* standard error, *CI* confidence interval

***P < 0.001

**Fig. 1:**
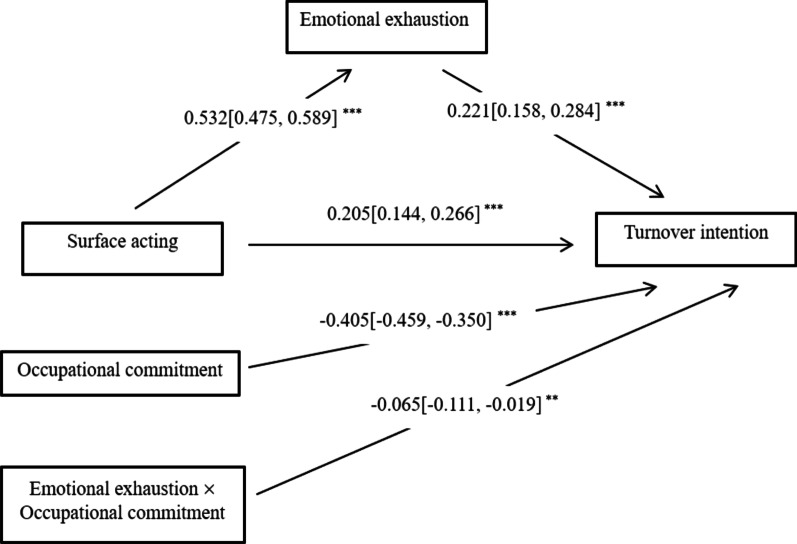
Statistical diagram of the moderated mediation model (*n* = 953). Covariates controlled (gender, age, marital status, and family yearly income) in the modeling analysis were found to be statistically insignificant, except for educational background which had a small positive influence on emotional exhaustion (*β* = 0.109, *P* = 0.041). 95% confidence intervals were in parentheses. **P < 0.01, ***P < 0.001

**Fig. 2:**
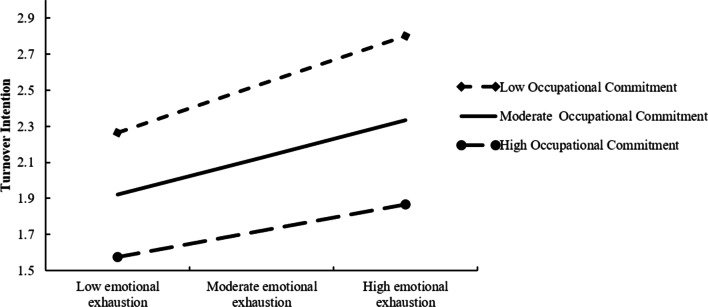
Interaction plot under the different conditions of occupational commitment

The result of the moderated mediation model is shown in Fig. 1. The interaction term (emotional exhaustion * occupational commitment) significantly predicted turnover intention (*β* = − 0.065, *P* < 0.01). Thus, occupational commitment moderated the effect of emotional exhaustion on turnover intention, as verified for H5. Figure 2 depicts the interaction plot for the relationship between emotional exhaustion and turnover intention under the condition of relatively low (mean − 1SD), moderate (mean), and relatively high (mean + 1SD) occupational commitment. As illustrated, the slope of the association between emotional exhaustion and turnover intention was steeper when the occupational commitment was low (simple slope = 0.288), while the slope was flatter when the occupational commitment was high (simple slope = 0.160). The association between emotional exhaustion and turnover intention was weaker when the occupational commitment was relatively high.

The bootstrapped method was used to test whether the indirect effect of surface acting on turnover intention through emotional exhaustion was moderated by occupational commitment, with the results shown in Table 5. The value of the index was − 0.035 (95% CI [− 0.058, − 0.010], not including 0, significant), indicating a significant conditional indirect effect of surface acting on turnover intention mediated by emotional exhaustion and moderated by occupational commitment, as verified for H6. The bootstrapped results also showed the indirect effect was conditional on different levels of occupational commitment: the indirect effect was stronger (*β* = 0.152, 95% CI = [0.102, 0.202]) when the occupational commitment was low, while the indirect effect was weaker (*β* = 0.083, 95% CI = [0.042, 0.124]) when the occupational commitment was high.

**Table 5:** Results of the moderated mediation model

Conditions of occupational commitment	Conditional indirect effect				Moderated mediation			
	Estimate	SE	95% confidence interval		Index	SE	95% confidence interval	
			Lower limit	Upper limit			Upper limit	Upper limit
Low	0.152	0.025	0.102	0.202	− 0.035	0.012	− 0.058	− 0.010
Moderate	0.117	0.019	0.080	0.155				
High	0.083	0.021	0.042	0.124				

## Discussion

The study aims to examine whether rural family doctors’ surface acting is associated with turnover intention through emotional exhaustion, and to further explore whether occupational commitment moderates this effect, and, more specifically, whether occupational commitment moderates the relationship between emotional exhaustion and turnover intention. The analysis results revealed that all the hypotheses were supported, in which emotional exhaustion partially mediated the effect of surface acting on turnover intention, and occupational commitment moderated the effect of emotional exhaustion on turnover intention and further moderated the mediating effect.

The study indicates that while surface acting is significantly and positively related to turnover intention, it has a stronger relationship with emotional exhaustion as a mediating factor. Deng, Wang et al. [52, 86] found a significant positive effect of surface acting on emotional exhaustion in a study with Chinese nurses. Kim’s meta-analysis with Korean nurses found that emotional exhaustion was an antecedent variable of turnover intention [56], and this finding was also confirmed by Gan’s study of factors influencing turnover intention among Chinese general practitioners [17]. Guo et al. [44] also indicated that surface acting was an antecedent variable of turnover intention. Moreover, Na [61] and He [32] verified the mediating role of emotional exhaustion between surface acting and turnover intention through Korean nurses and Chinese employees, respectively. These findings are consistent with ours, which indicated that “surface acting leads to emotional exhaustion, which in turn causes turnover intention”, and can be further validated by the idea of “loss cycle” in the COR theory, where surface acting is a potential threat to emotional resources and self-protective defensive mechanisms are motivated by continuous resource depletion. As family doctors are constantly required to perform emotional work and surface acting to provide better services for rural residents, they may experience a loss of resources and go through emotional exhaustion if their efforts are not rewarded as expected. Considering the lack of resources, rural family doctors protect and prioritize remaining resources and therefore may be prone to turnover.

Furthermore, the negative relationship between occupational commitment and emotional exhaustion with turnover intention has been well established in related studies, but the moderating role of occupational commitment in the process has not been fully explored [16, 62, 66]. In this study, occupational commitment has been found to have an important moderating role in the relationship between emotional exhaustion and turnover intention via literature review, theoretical derivation, and empirical validation. High levels of occupational commitment weaken the effect of emotional exhaustion on turnover intention among rural family doctors. When emotional exhaustion persists at work, rural family doctors with high occupational commitment are less likely to have turnover intention than those with low occupational commitment. In addition, rural family doctors with low occupational commitment may not be able to obtain or regain sufficient resources to weaken the adverse effects resulting from emotional exhaustion. Consistent with the resource investment principle of the COR theory, occupational commitment can promote rural family doctors to enhance and preserve their inner resources, minimize the depletion degree of their emotional resources and regain the expected resources. The study also confirms that occupational commitment moderates the mediating effect of surface acting on turnover intention via emotional exhaustion, and deepens our understanding of how occupational commitment weakens the adverse consequences of emotional exhaustion.

### Theoretical implications

The first contribution of this study is the introduction of emotional labor strategies into the research on family doctors, which provides a more comprehensive understanding of the work characteristics of family doctors by making a breakthrough from existing perspectives that medical services require both mental and physical effort. Emotional labor is also an important form of labor for family doctors in their regular services. In this study, emotional dissonance theory is applied to explain the effect of surface acting on turnover intention. As family doctors’ inner feelings and emotional displays are not consistent when engaging in surface acting, they are prone to emotional dissonance, and, in order to banish this uncomfortable experience, they are more likely to have turnover intention.

The second theoretical contribution of this study is to clarify the mediating role of emotional exhaustion in the effect of surface acting on turnover intention using the COR theory and to further explore the moderating role of occupational commitment. Following the COR theory, the stressful circumstances associated with surface acting can deplete rural family doctors’ emotional resources, leading to emotional exhaustion. When emotional resources are consistently depleted, self-protective defensive mechanisms are motivated to encourage individuals to conserve and prioritize their remaining resources, thus halting investment and activating turnover intention. However, if resources are able to be replenished when rural family doctors experience emotional exhaustion, the effect of emotional exhaustion on turnover intention is weakened. Occupational commitment can provide rural family doctors with inner resources, such as self-efficacy and resilience.

### Practical implications

Chau et al. [41] emphasized that individuals engaging in surface acting and experiencing emotional exhaustion may not necessarily be undesirable employees who have to be turnover. Rather, they are possibly excellent employees who are merely adopting a self-protecting strategy of turnover to prevent their remaining resources from being insufficient for appropriate emotional displays. Additionally, Chau et al. [41] further stated that surface acting affects turnover intention through the process of emotional exhaustion, which implies a period of evolution from surface acting to turnover intention, and organizations should take full advantage of this time to adopt targeted emotional labor interventions to minimize the adverse consequences of surface playing and emotional exhaustion, and retain excellent employees. Therefore, we propose the following practical implications.

Organizations can train family doctors to use various emotion management strategies in their healthcare delivery. Engagement strategies that direct the family doctors’ attention or efforts to cope with current emotions and challenges using reassessment and organizational support can be adopted. Diversion strategies that divert attention, such as avoidance, can also be used. It confirms that individuals being instructed to use engagement strategies respond with more positive emotions and better abilities to meet emotional demands [87]. It is recommended that priority be given to training family doctors with engagement strategies, and that family doctors actively use deep acting rather than surface acting to be empathetic with rural residents. If engagement strategies contribute to burnout for family doctors, a short-term diversion strategy can be used to minimize the use of surface acting. In addition, the publicity of the family doctor contract services system should be strengthened to increase rural residents’ familiarity with the services provided by family doctors, improve residents’ cooperation, and reduce the depletion of emotional resources caused by the frequent surface acting of family doctors.

The other aspect requires reinforcing the occupational commitment of family doctors, replenishing the resources needed for their work, and minimizing the possibility of emotional exhaustion and its adverse consequences. It is important to strengthen the pre-service education of family doctors to improve their enthusiasm and beliefs about what they are doing and to increase personal characteristic resources such as self-efficacy and resilience. At the same time, effort should also be made to provide external support for the work of family doctors, especially in rural areas. It is essential to accelerate the construction of a grassroots talent pool and increase investment in basic supplies, which can help replenish resources in time and avoid turnover intention when family doctors’ resources are depleted.

## Limitations and future research

Despite the strengths of this study, there are some limitations. First, we did not incorporate individual differences and organizational factors into the hypothesized model. In the future, we could incorporate individual factors such as demographic information (e.g., gender, age), and psychological and personality traits into the study regarding the effect of surface acting on turnover intention. In addition, we could explore interventions to weaken the adverse effects of emotional exhaustion at the organizational level, such as organizational support and organizational identity. Second, the cross-sectional nature of this study means that it is impossible to demonstrate causal relationships among the variables. More importantly, the variables involved in the study, namely surface acting, emotional exhaustion, turnover intention, and occupational commitment, are of a dynamic and temporal nature and require a longitudinal study to better capture the information. Future studies could take advantage of techniques, such as the experience sampling method, to allow participants to repeat assessments at various points in time, in order to obtain a better understanding of the fluctuations in family doctors’ turnover intention and influencing factors. Third, the data were collected using a self-reporting approach, with social desirability effects due to observation bias being unavoidable. In future studies, we would consider designing questionnaires from multiple perspectives, including immediate leaders, team colleagues, and rural residents, to investigate family doctors’ turnover intention and influencing factors. Finally, the participants in this study were mainly from Shandong Province, an economically developed province in eastern China, and may not be representative of the current status of family doctors in all rural areas of China. In future studies, we could sample one province in central China and one province in western China to represent the economically moderate and underdeveloped provinces, respectively, in order to increase the generalizability of the findings to a larger population.

## Conclusions

This study, conducted with family doctors in rural China, confirms that emotional exhaustion partially mediates the relationship between surface acting and turnover intention, and that occupational commitment moderates the direct effect of emotional exhaustion on turnover intention and further moderates the mediating effect. In light of the findings of this study, it is suggested that engagement and diversion strategies be used to respond to emotional labor demands and to reduce the practice of surface acting by family doctors. In addition, pre-service education and external resources should be provided to increase family doctors’ occupational commitment and minimize the incidence of emotional exhaustion and turnover intention.

## Data Availability

The datasets used and/or analyzed during the current study are available from the authors on reasonable request.
